# Tuberculosis of the Breast: An Initial Presentation of the Metabolic Syndrome with Type 2 Diabetes Mellitus in a Young Nigerian Woman

**DOI:** 10.1155/2016/5485862

**Published:** 2016-03-10

**Authors:** M. A. Adeiza, R. Yusuf, A. A. Liman, P. Abur, F. Bello, A. A. Abba

**Affiliations:** ^1^Pulmonology Unit, Department of Medicine, Ahmadu Bello University Teaching Hospital, PMB 06, Shika, Zaria, Nigeria; ^2^Department of Chemical Pathology, Ahmadu Bello University Teaching Hospital, PMB 06, Shika, Zaria, Nigeria; ^3^Department of Histopathology, Ahmadu Bello University Teaching Hospital, PMB 06, Shika, Zaria, Nigeria; ^4^Department of Surgery, Ahmadu Bello University Teaching Hospital, PMB 06, Shika, Zaria, Nigeria; ^5^Endocrine Unit, Department of Medicine, Ahmadu Bello University Teaching Hospital, PMB 06, Shika, Zaria, Nigeria

## Abstract

Breast tuberculosis is an uncommon presentation of extra pulmonary tuberculosis. A 40-year-old obese woman presented with a right breast abscess which had failed to heal after surgical drainage. There was no family history of breast disease. Biopsy and histology of the lesion showed chronic granulomatous inflammation with positive stains for acid fast bacilli compatible with tuberculosis. Further evaluation confirmed metabolic syndrome with type 2 diabetes mellitus. She was placed on antituberculosis chemotherapy and appropriate therapy for diabetes mellitus with complete resolution of the lesion. We report this case because of its rarity and to highlight the association between tuberculosis an infectious disease and overnutrition in diabetes mellitus, a noncommunicable disease.

## 1. Introduction

Tuberculosis (TB) is a major public health problem in developing countries of sub-Saharan Africa and Asia. According to the WHO global TB report, there were 9 million cases of tuberculosis in 2014 [1]. The TB epidemic is fueled by immunosuppression especially HIV, but, recently, diabetes mellitus has been recognized as an important risk factor and the two epidemics are set to converge [2, 3] this time with the epidemic of a noncommunicable disease fuelling an epidemic of an infectious disease. As rural societies in developing countries westernize and lifestyle and eating habits change, it is projected that 366 million people will have diabetes mellitus by 2030 with most of them living in Africa and Asia [4]. The International Diabetic Federation estimates that a quarter of the world's adult population has the metabolic syndrome [5]. Metabolic syndrome is a state of chronic low grade inflammation as a consequence of a complex interplay between genetic and environmental factors. Insulin resistance, visceral adiposity, atherogenic dyslipidemia, endothelial dysfunction, genetic susceptibility, elevated blood pressure, hypercoagulable state, and chronic stress are the several factors which constitute the syndrome [6].

We present a case of breast tuberculosis in a Nigerian woman who presented with a breast abscess that is thought to be pyogenic initially, but, on histology, it was found to be tuberculosis of the breast with the metabolic syndrome and type 2 diabetes mellitus. We highlight this emerging interaction between the two conditions and proffer solutions for control.

## 2. Case Report

A 40-year-old woman was referred from a peripheral hospital to our clinic with right breast abscess which ulcerated and failed to heal despite incision, drainage, and antibiotics for 4 weeks.

Her problem started 4 weeks earlier when she noticed a swelling in the lower outer quadrant of her right breast. Within a week, it gradually increased in size and became painful. By the second week, it ruptured and began to discharge purulent material. There was no swelling elsewhere, but the patient had developed a low grade intermittent fever. There was no cough, weight loss, or drenching night sweats. She was not breastfeeding and there was no galactorrhoea or nipple discharge.

There was no known risk factor for tuberculosis and no family history of breast disease. She did not use hormonal contraceptives and gynaecological history was unremarkable. She was para^2+0^ (2 alive) with last child birth being 3 years previously and history of macrosomic babies. She had never smoked cigarette nor ingested alcohol and was not known to be hypertensive or diabetic and had no family history of similar illness. However, further probing highlighted a poor eating habit over the years and a lifestyle that was sedentary.

Physical examination revealed a well preserved but morbidly obese middle aged woman who was not pale or febrile. There were no significant peripheral lymphadenopathy, corneal arcus, or xanthelasma. She however had skin tags around the neckline but no acanthosis nigricans. Anthropometric measurements were height of 160 cm, weight of 115 kg, and body mass index (BMI) of 44.9 kg/m^2^. Waist circumference was 138 cm with a waist-to-hip ratio of >1.0.

**Figure 1:**
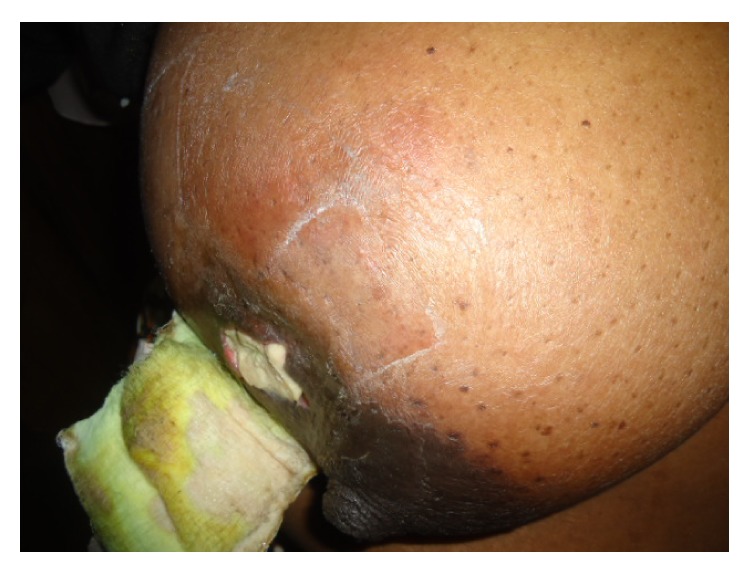
Tuberculous abscess in lower outer quadrant of right breast with erythema and peau d'orange.

Breast examination showed an irregularly shaped 6 cm × 4 cm ulcer in the lower outer quadrant of the right breast. The edge was undermined and base showed yellowish slough. Surrounding skin was erythematous with peau d'orange (Figure 1). No axillary lymphadenopathy was detected. Chest examination was normal and blood pressure (BP) was 180/110 mmHg sitting with normal heart sounds. There were no significant abdominal and nervous system findings.

Differential diagnoses were pyogenic breast abscess and tuberculosis of the breast on a background of metabolic syndrome to exclude carcinoma of the breast. Breast ultrasound scan showed irregular inflammatory thickening of the glandular tissue at the right periareolar area. The affected area measured 5.2 × 3.9 cm. There were no ductal dilatation and no involvement of the pectoralis muscles.

**Figure 2:**
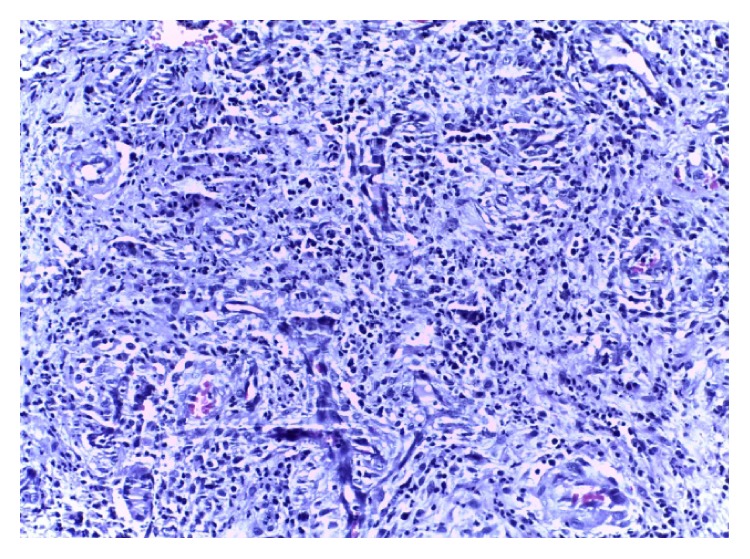
Tuberculous granuloma, HE ×40.

**Figure 3:**
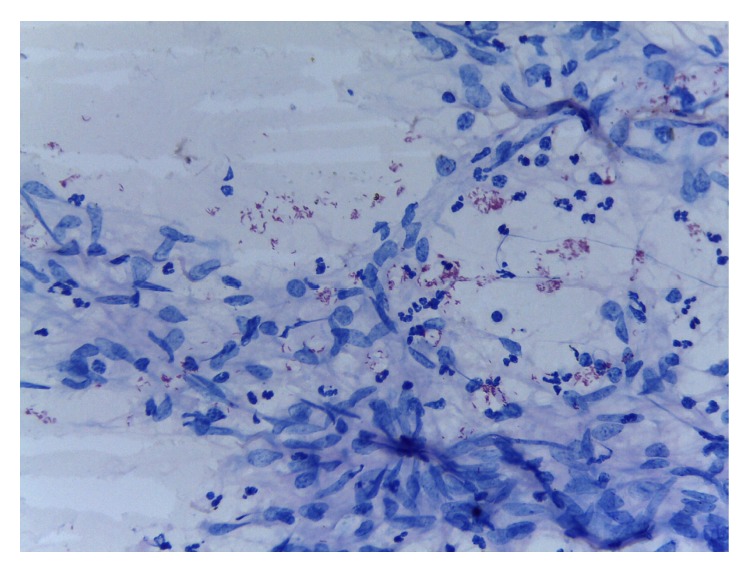
Special stain (ZN) for AFB ×100.

Biopsy and histology of the ulcer (Figure 2) showed fragments of fibroadipocytic connective tissue exhibiting intense inflammation with neutrophil polymorphs, lymphoplasma cells, and epithelioid histiocytes. Areas of fibrosis and focal fat necrosis were noted. The overall features were those of diffuse granulomatous reaction and special stain was positive for acid fast bacilli (Figure 3). The tuberculin skin test reaction was 10 mm and sputum AFB microscopy was negative. Fasting plasma glucose (FPG) was 16.0 mmol/L, while two-hour postprandial glucose (2hrPP) was 21.0 mmol/L and HbA1C was 11.0%. Triglyceride and high-density lipoprotein (HDL) cholesterol were 53 mg/dL and 58 mg/dL, respectively. Chest X-ray was normal with no lung parenchymal or bony chest wall involvement.

Based on the histological findings and metabolic derangement, a diagnosis of tuberculosis of the right breast with the metabolic syndrome was made. She was started on anti-TB chemotherapy: tabs of rifampicin 750 mg daily, tabs of isoniazid 375 mg daily, tabs of ethambutol 1.4 mg daily, and tabs of pyrazinamide 2 g daily in a fixed dose combination. She also had tabs of metformin 1 g twice daily, tabs of glimepiride 2 mg daily, and tabs of lisinopril 5 mg daily with education on diet and a healthy lifestyle. The lesion healed with complete resolution in 6 months. She was discharged to be followed up in the endocrine and metabolic clinic with a FPG of 3.8 mmol/L and 2hrPP of 5.7 mmol/L.

## 3. Discussion

Tuberculosis of the breast is a rare form of extrapulmonary tuberculosis (EPTB). It was first described by Cooper in 1829 who called it “scrofulous swelling of the bosom” [7]. It accounted for 0.6% and 0.7% of histologically diagnosed breast lumps in Enugu and Maiduguri in Nigeria, respectively [8, 9]. The breast is inherently resistant to primary breast TB except around the time of lactation or breastfeeding [10]. Various theories have been proposed to explain why the incidence rises around this period, that is, increased blood flow and abrasion in the skin or through ducts at the nipples [11]. In this index case, no such local risk factor was found, prompting the search for a systemic illness that may constitute a risk factor for TB. Our patient was HIV negative but morbidly obese and further evaluation confirmed that she had metabolic syndrome with type 2 diabetes.

The interaction between diabetes mellitus and tuberculosis has already been described [12, 13], but this is a rare presentation of tuberculosis of the breast in association with the metabolic syndrome. The metabolic syndrome refers to a well-defined group of risk factors, including central obesity and inflammation, for the development of diabetes and cardiovascular disease [14]. According to the WHO [15], the metabolic syndrome is diagnosed when a patient has insulin resistance defined as type 2 diabetes mellitus (DM) or impaired fasting glucose (IFG) (>100 mg/dL) or impaired glucose tolerance (IGT), plus two of the following:

- abdominal obesity with waist-to-hip ratio >0.9 in men or >0.85 in women, or BMI > 30 kg/m^2^;
- triglycerides ≥ 150 mg/dL and/or HDL-cholesterol < 40 mg/dL in men and <50 mg/dL in women;
- BP ≥ 140/90 mmHg;
- microalbuminuria ≥ 20 *μ*g/min or albumin-to-creatinine ratio ≥ 30 mg/g.

In the literature, evidence of the link between the metabolic syndrome and infections like HIV and chronic hepatitis C virus infection exists [16, 17], but no such link has been established for TB with TB as a chronic debilitating illness generally considered to be associated with undernutrition as opposed to overnutrition [13].

The clinical presentation of tuberculosis of the breast is usually nonspecific, and after evaluation the differential diagnosis can range from breast abscess to carcinoma of the breast [18]. It must also be noted that TB of the breast usually coexists as a secondary component in a disseminated disease to a primary focus usually in the lung parenchyma or bony chest wall [19]. In this patient, however, the presentation was primary breast TB as no other organ was found to be involved in the disease process after her evaluation. This patient was initially thought to have pyogenic breast abscess, but it failed to heal with antibiotics. Biopsy and histological examination revealed the correct diagnosis. She was started on standard antituberculosis chemotherapy with a good response to treatment at 6 months.

## 4. Conclusion

Breast TB is an unusual presentation of EPTB. The interaction with the metabolic syndrome and diabetes mellitus means that the two epidemics are converging. We recommend routine screening of all TB patients for metabolic syndrome and diabetes mellitus and emphasize the role of public health measures like good nutrition and lifestyle modifications to control a noncommunicable disease that is set to fuel the tuberculosis pandemic.
